# Supplementary material for: Acceptance and compliance with micronutrient powder (MNP) among children aged 6–23 months in northern Nigeria
Source: PLOS Glob Public Health. 2022 Oct 17;2(10):e0000961. doi: 10.1371/journal.pgph.0000961 (PMC10022258; doi:10.1371/journal.pgph.0000961)
Supplement: S1 Dataset — (ZIP) [file pgph.0000961.s008.zip › S8_Dataset/Spot Checks.zip!/Spot Checks/030715_K_BK_AD_2_SC.a.pdf]

# SPOT CHECK RECORDING FORM

**State: Kebbi LGA: Birnin kebbi Community: Nufawa**

**Sex of index child: F   Age of index child (in months): 10 months**

## Spot Check Information

1. **Date of MNP distribution to household** (circle option below based on LGA name above)**:**
   - **Kebbi:** BirninKebbi&Jega LGA: **June 16**
   - **Kebbi:** Danko-wasagu: **June 17**
   - **Adamawa:**Song, Ganye: **June 18**
   - **Adamawa:**Guyuk **June 19**
2. **Date of Spot Check Observation: 03/07/15**
3. **Number of sachets remaining**(Ask the caregiver to show you and count the specific number of sachets remaining)**:**

   45

4. **Number of sachets that <u>should remain</u>using one MNP sachet per day** (Analyst only)**:**

   44

5. **Number of sachets remaining / number of sachets should be remaining**(Analyst only)**:**

   **_____45____ / _____44____ = ___102.3______ %**

## Probes

**Question 1.** You have **XX** MNP sachets remaining. Explain how easy or difficult it was to use the MNP product on a daily basis for your child 6 – 23 months? (Circle one answer)

**Very Difficult** **Difficult** **Easy** **Very Easy**

**Question 2.**Describe any specific challenges that you faced that made the MNP difficult to use on a daily basis?
The sachet is very difficult to open it was advice to use hand and open the sachet

**Question 3.** Describe specific aspects of the MNP product that you or your child enjoyed that made it easier to use on a daily basis?
I like the product because it has improved her health. It has also improved her appetite greatly

**Question 4.** What questions do you have about the MNP based on your experience using it?
I do not have any

**Question 5.** What specific recommendations do you have that we can do as a program to make the MNP easier to use on a daily basis?
